# A novel chemically defined medium for the biotechnological and biomedical exploitation of the cell factory *Leishmania tarentolae*

**DOI:** 10.1038/s41598-024-60383-1

**Published:** 2024-04-26

**Authors:** Giulia Maria Cattaneo, Ilaria Varotto-Boccazzi, Riccardo Molteni, Federico Ronchetti, Paolo Gabrieli, Jairo Alfonso Mendoza-Roldan, Domenico Otranto, Emanuele Montomoli, Claudio Bandi, Sara Epis

**Affiliations:** 1 Department of Biosciences, University of Milan, 20133 Milan, Italy; 2 Pediatric CRC ‘Fondazione Romeo ed Enrica Invernizzi’, University of Milan, 20157 Milan, Italy; 3 Department of Veterinary Medicine, University of Bari, 70010 Valenzano, Italy; 4 Department of Veterinary Clinical Sciences, City University of Hong Kong, Hong Kong, Republic of China; 5 Department of Molecular and Developmental Medicine, University of Siena, 53100 Siena, Italy; 6 VisMederi, 53100 Siena, Italy

**Keywords:** *Leishmania* culture, *Leishmania tarentolae*, Chemically defined medium, Protein expression, Promastigotes, Cell factory, Microbiology, Non-model organisms, Assay systems

## Abstract

The development of media for cell culture is a major issue in the biopharmaceutical industry, for the production of therapeutics, immune-modulating molecules and protein antigens. Chemically defined media offer several advantages, as they are free of animal-derived components and guarantee high purity and a consistency in their composition. Microorganisms of the genus *Leishmania* represent a promising cellular platform for production of recombinant proteins, but their maintenance requires supplements of animal origin, such as hemin and fetal bovine serum. In the present study, three chemically defined media were assayed for culturing *Leishmania tarentolae*, using both a wild-type strain and a strain engineered to produce a viral antigen. Among the three media, Schneider's *Drosophila* Medium supplemented with Horseradish Peroxidase proved to be effective for the maintenance of *L. tarentolae* promastigotes, also allowing the heterologous protein production by the engineered strain. Finally, the engineered strain was maintained in culture up to the 12th week without antibiotic, revealing its capability to produce the recombinant protein in the absence of selective pressure.

## Introduction

*Leishmania tarentolae* is a kinetoplastid protozoon belonging to the subgenus *Sauroleishmania*^1^. This species is associated with reptiles, especially lizards (Sauria), is not pathogenic to mammals, including humans, and is characterized by having a rapid growth rate in in vitro culture and low maintenance costs, resulting in an excellent candidate for biotechnological applications^2,3^.

Commonly, *Leishmania* parasites are maintained in vitro as promastigotes, exploiting a liquid medium (e.g., RPMI 1640 medium, Schneider’s insect medium, M199, DMEM and BHI) supplemented with antibiotics, sera of animal origin (e.g. fetal bovine serum—FBS, fetal calf serum—FCS), and, in some cases, other components such as l-glutamine, hemin and urine^4–11^. These components provide the required nutrients not included in a classic medium and lead to growth at high cell densities, with the possibility of a scaling-up. Besides supplying the required nutrients for *L. tarentolae* cell growth, the use of animal serum entails some disadvantages. For example, FBS is one of the most expensive supplements for cell media and it is easily contaminated if not properly managed. Regarding the ethical perspective, it should also be considered how FBS collection occurs in bovine fetuses and how its demand is unsustainable for a global supply^12–14^. Furthermore, it is highly recommended that the use of animal-derived compounds and fluids is minimized, and possibly avoided, in cell lines maintenance, in the area of biopharmaceutical applications^12,13^. The use of chemically-defined media is thus recommended in these contexts.

Moreover, one of the main drawbacks associated with the use of animal-derived substances is the potential presence of viruses or contaminating microorganisms, that could then be incorporated into the final product^13^.

Media that are not derived from animals have already been used in the maintenance of different *Leishmania* species, as alternatives to the BHI medium. These include RPMI-1640, Dulbecco’s Modified Eagle Medium (DMEM), Luria–Bertani (LB) broth and Schneider’s *Drosophila* Medium^5,6,8,9,14–17^*.* However, as these media require the addition of components of animal origin (e.g., sera or hemin), finding alternative supplements to them has turned out to be difficult to achieve^14^. Indeed *Leishmania* spp., like other hemoflagellates, are obligate parasites, adapted to grow inside specific hosts, obtaining specific cofactors and nutrients, among which iron. For the in vitro growth of *Leishmania* spp. This micronutrient is usually added in the form of iron-containing porphyrins, like heme, or through the animal serum, such as FBS or FCS^18,19^. The addition of this type of supplementation is mandatory for in vitro* Leishmania* maintenance: its absence not only inhibits *Leishmania* cell multiplication, but also leads to cell death. A pioneer study by Gaughan and Krassner^18^ identified two iron porphyrins catalase and peroxidase as promising alternatives to hemin, in order to supplement in BHI medium^18^. A soy protein isolate (SPI) was also identified as a possible alternative to FBS, for the culturing of *Leishmania donovani* promastigotes in RPMI-1640^14^.

Our aim was to optimize an animal-free medium for the maintenance of *L. tarentolae* promastigotes. To this purpose we tested different media, with different types of supplementations, determining growth parameters at different conditions. Considering the potential applications of *L. tarentolae* in the biothechnological and biopharmaceutical areas, we also determined the production of a recombinant protein by an engineered strain of the parasite.

## Results and discussion

### Adaptation of *Leishmania* strains to RPMI-1640 Medium and Schneider’s *Drosophila* Medium + soy protein isolate

The adaptation of *Leishmania* to different media was verified by evaluating cell growth and the morphology at the promastigote stage. Firstly, we tested the RPMI and Schneider media, adding the SPI, following the DA protocol. As positive control, cells were grown respectively in RPMI and Schneider media supplemented with FBS. Both the SPI-supplemented media did not allow to obtain an effective culture of Lt-P10: promastigote cells appeared rounded, differently from cells maintained in the commonly used media (RPMI + FBS; Schneider + FBS), where cells display a normal, elongated promastigote morphology (Supplementary File 1A). In addition, the number of cells that were counted in the SPI-supplemented media at day 4 was visibly reduced compared to that after the inoculum. In summary, results show that the first two media that were tested are not suitable for *L. tarentolae* maintenance, at least in the present experimental setting.

As previously reported, our strategy was to proceed with SA only after achieving an at least partial success with DA. Similarly, the adaptation of the engineered Lt-RBD was performed only with media that resulted suitable for the growth of the wild type strain Lt-P10. Consequently, we did not continue the experiments with RPMI + SPI or Schneider + SPI.

### Schneider’s *Drosophila* Medium + Horseradish Peroxidase: sequential adaptation strategy

Schneider + HRP was successfully assayed in DA for the maintenance of both Lt-P10 and Lt-RBD. Therefore, both strains were then subjected to SA, using BHI as the control medium. For a proper comparison, the same number of cells was inoculated in the flasks with the two media (BHI; Schneider + HRP) and the number of cells/ml was monitored twice a week by optical microscopy. The experiment was repeated three times. In all replicas we observed a reduction in the number of promastigotes in Schneider + HRP once we reached a medium in which the serum was absent, with complete replacement by HRP (Table 1). In order to present the progress in cell adaptation, Table 1 shows the mean number of cells/ml for a single replica.

**Table 1:** Cell density mean (No cells/ml) and standard deviation (Std. Dev.) of Lt-P10 and Lt-RBD strains in Schneider's *Drosophila* Medium following the sequential adaptation protocol (SA), from a 100% serum supplemented medium (SSM, Schneider + 10% FBS) to a 100% serum free medium (SFM, Schneider + HRP). BHI medium was used as reference.

No passages	BHI				Schneider + HRP (SA protocol)				SA step
	Lt-P10		Lt-RBD		Lt-P10		Lt-RBD		
	No cells/ml	Std. Dev.	No cells/ml	Std. Dev.	No cells/ml	Std. Dev.	No cells/ml	Std. Dev.	
1	8.5 × 10^7^	2.12 × 10^7^	1.85 × 10^8^	7.78 × 10^7^	1.65 × 10^8^	4.95 × 10^7^	7.50 × 10^7^	7.07 × 10^6^	80% SSM 20% SFM
2	1.51 × 10^8^	6.36 × 10^6^	1.51 × 10^8^	6.36 × 10^6^	1.40 × 10^7^	2.83 × 10^6^	6.30 × 10^7^	1.7 × 10^7^	80% SSM 20% SFM
3	1.49 × 10^8^	2.76 × 10^7^	1.36 × 10^8^	3.54 × 10^6^	6.65 × 10^7^	3.54 × 10^6^	8.15 × 10^7^	1.91 × 10^7^	80% SSM 20% SFM
4	1.63 × 10^8^	2.12 × 10^6^	1.17 × 10^8^	1.84 × 10^7^	8.72 × 10^7^	1 × 10^8^	1.90 × 10^8^	2.12 × 10^6^	50% SSM 50% SFM
5	1.76 × 10^8^	1.98 × 10^7^	1.03 × 10^8^	2.33 × 10^7^	1.35 × 10^7^	3.54 × 10^6^	3.50 × 10^7^	0	50% SSM 50% SFM
6	1.80 × 10^8^	5.66 × 10^7^	2.10 × 10^8^	8.49 × 10^7^	3.05 × 10^7^	4.95 × 10^6^	6 × 10^5^	0	50% SSM 50% SFM
7	1.70 × 10^8^	1.63 × 10^7^	2.10 × 10^8^	1.41 × 10^7^	5.2 × 10^7^	7.07 × 10^6^	3.95 × 10^7^	4.95 × 10^6^	20% SSM 80% SFM
8	2.50 × 10^8^	8.49 × 10^7^	2.60 × 10^8^	7.07 × 10^7^	1.48 × 10^8^	6.36 × 10^6^	8.05 × 10^7^	2.33 × 10^7^	20% SSM 80% SFM
9	1.40 × 10^8^	4.24 × 10^7^	2.40 × 10^8^	0	1.7 × 10^8^	2.38 × 10^6^	2.70 × 10^8^	9.9 × 10^7^	20% SSM 80% SFM
10	2 × 10^8^	1.13 × 10^8^	4.95 × 10^8^	3.54 × 10^7^	2.10 × 10^7^	2.83 × 10^6^	3.20 × 10^8^	8.49 × 10^6^	100% SFM
11	1.08 × 10^8^	1.06 × 10^7^	6.85 × 10^7^	2.19 × 10^7^	5 × 10^4^	4.24 × 10^4^	4 × 10^4^	0	100% SFM
12	2.7 × 10^8^	2.12 × 10^8^	1.55 × 10^8^	7.07 × 10^6^	3.15 × 10^4^	3.54 × 10^4^	6.50 × 10^4^	2.12 × 10^4^	100% SFM
13	1.17 × 10^8^	1.34 × 10^7^	1.50 × 10^8^	1.70 × 10^7^	9.5 × 10^4^	2.12 × 10^4^	4 × 10^4^	2.83 × 10^4^	100% SFM

Lt-P10 and Lt-RBD promastigotes in Schneider + HRP were morphologically similar to the respective controls in BHI, presenting the form of elongated nectomonads, short nectomonads or metacyclic promastigotes (Supplementary File 1B). After maintenance in Schneider + HRP, *Leishmania* cells displayed limited motility, in some cases limited to flagellar movement. In contrast, both Lt-P10 and Lt-RBD in BHI displayed active motility.

Once moved to a 100% serum free medium (Schneider + HRP), the number of cells/ml decreased in both Lt-P10 and Lt-RBD (Table 1, passage 10–13). Considering the low number of cells in Schneider + HRP cultures, from passage 10 (100% SFM), all the available cells were inoculated into a new medium for the next passage. Here, the number of cells/ml in Lt-P10 cultured in Schneider + HRP, decreased from 2.10 × 10^7^ cells/ml (passage 10, Table 1) to 9.5 × 10^4^ cells/ml (passage 13, Table 1). Similarly, in Lt-RBD cultured in Schneider + HRP, the number of cells/ml reduced from 3.20 × 10^8^ cells/ml (passage 10, Table 1) to 4 × 10^4^ cells/ml (passage 13, Table 1). Lt-P10 and Lt-RBD were therefore unable to replicate and survive once moved in Schneider + HRP.

The SA protocol has already been applied in several studies, in different types of microorganisms and cells, thanks to some advantages, such as progressive adaptation to the new medium and minimal stress for the cells^20–23^. In our SA protocol, each step in serum reduction requires 3 passages in the new medium. Therefore, to achieve an adaptation to a medium with 80% reduction of the serum, approximately 20 days are required (Table 1). In addition, to obtain the culturing in a serum-free medium, which is anyway not satisfactory, 30 days are required. In conclusion, these results, in agreement with the microscopical observations, indicate that maintenance of *L. tarentolae* in a completely chemically defined medium such as Schneider + HRP is not only difficult (or impossible) to achieve, but also time consuming.

### Schneider's *Drosophila* Medium + Horseradish Peroxidase: direct adaptation strategy

Lt-P10 and Lt-RBD strains were successfully maintained the HRP-supplemented media, displaying an unaltered morphology compared with the same strains maintained in the control medium (BHI) (see below). The adapted Lt-P10 and Lt-RBD strains were then stored at − 80 °C. Then, a frozen Lt-RBD stock was reactivated in FBS-supplemented medium, readapted to the Schneider + HRP following a DA protocol, and assayed in comparison with un-frozen Lt-P10 and Lt-RBD, always in Schneider + HRP (control: reactivated Lt-RBD in Schneider + FBS).

**Figure 1:**
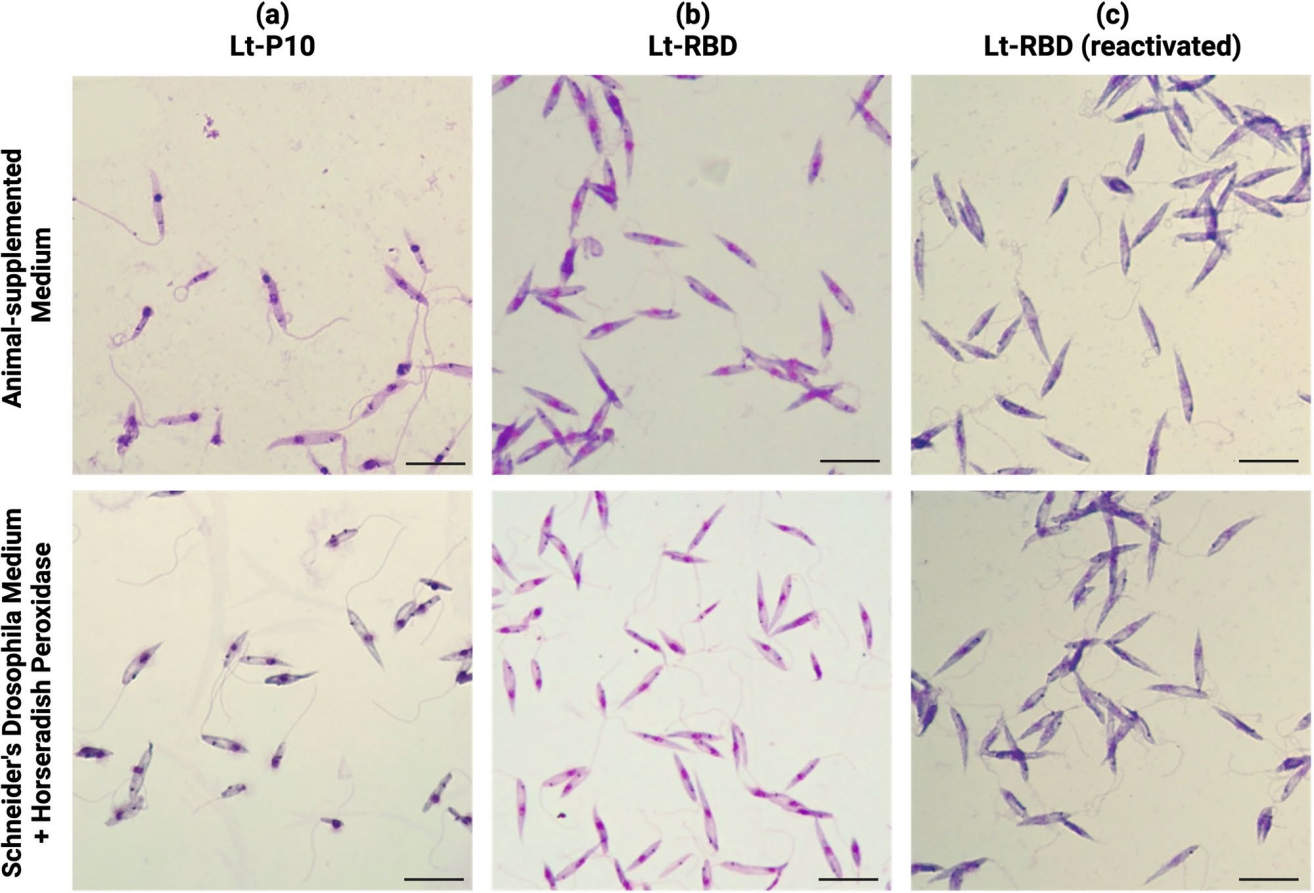
Morphological evaluation of Lt-P10, Lt-RBD and the reactivated Lt-RBD promastigotes 96 h after the inoculum in Schneider HRP, and in the media supplemented with animal components. Giemsa staining of: (**a**) Lt-P10 cultured in BHI and in Schneider + HRP (DA); (**b**) Lt-RBD promastigotes cultured in BHI medium and in Schneider + HRP (DA); (**c**) The reactivated Lt-RBD promastigotes cultured in Schneider + 10% FBS and in Schneider + HRP (DA); Scale bar: 10 µm.

During the 5 days, cells in Schneider + HRP resulted to be elongated nectomonads, short nectomonads and metacyclic promastigotes, such as in the conventional media (Fig. 1).

During the week, *Leishmania* cells displayed a similar motility in the chemically defined medium and in the control media, for all the three strains considered (Lt-P10, Lt-RBD and the reactivated Lt-RBD). At the beginning and at the end of the week, *Leishmania* cells resulted to be in the stationary phase of the curve while in the middle of the week, cells were in the logarithmic phase. Coherently with the above observation, at the beginning and at the end of the week cells resulted to be more active (moving cells or static cells with active flagellum) than in the middle of the week (static with active flagellum).

Starting from a cell density of 3.2 × 10^6^ cells/ml, the three strains resulted to replicate successfully in Schneider + HRP (0.40 mg/ml). The cell density, starting from 3.2 × 10^6^ cells/ml, increased 8.3 times (2.67 × 10^7^ cells/ml), 9.3 times (2.98 × 10^7^ cells/ml) and 12.6 times (4.02 × 10^7^ cells/ml), respectively for Lt-P10, Lt-RBD and the reactivated Lt-RBD resulted (Table 2).

**Table 2:** Cell density mean (N° cells/ml), standard deviation (Std. Dev.) and growth rate of Lt-P10, Lt-RBD and the reactivated Lt-RBD strains during a week in Schneider's + HRP, during direct adaptation and in the conventional media (BHI and Schneider + FBS).

Hours		0	24	48	72	96	Growth rate
Lt-P10 BHI	No cells/ml	3.2 × 10^6^	1.51 × 10^7^	1.09 × 10^8^	1.8 × 10^8^	2.02 × 10^8^	63.13
	Std. Dev.	0	4.84 × 10^6^	5.16 × 10^7^	5.66 × 10^7^	3.19 × 10^7^	
Lt-P10 DA	No cells/ml	3.2 × 10^6^	8.78 × 10^6^	1.93 × 10^7^	2.17 × 10^7^	2.67 × 10^7^	8.3
	Std. Dev.	0	1.76 × 10^6^	6.92 × 10^6^	1.50 × 10^7^	1.28 × 10^7^	
Lt-RBD BHI	No cells/ml	3.2 × 10^6^	1.26 × 10^7^	7.83 × 10^7^	2 × 10^8^	3.22 × 10^8^	100.6
	Std. Dev.	0	5.25 × 10^6^	3.74 × 10^7^	8.81 × 10^7^	1.20 × 10^8^	
Lt-RBD DA	No cells/ml	3.2 × 10^6^	5.33 × 10^6^	1.92 × 10^7^	1.42 × 10^7^	2.98 × 10^7^	9.3
	Std. Dev.	0	1.31 × 10^6^	6.27 × 10^6^	7.70 × 10^6^	2.63 × 10^7^	
React LT-RBD SCHN + FBS	No cells/ml	3.2 × 10^6^	1.5 × 10^7^	6.1 × 10^7^	6.67 × 10^7^	1.59 × 10^8^	49.7
	Std. Dev.	0	9.59 × 10^6^	3.16 × 10^7^	2.25 × 10^7^	1.01 × 10^8^	
React Lt-RBD DA	No cells/ml	3.2 × 10^6^	7.75 × 10^6^	3.03 × 10^7^	2.7 × 10^7^	4.02 × 10^7^	12.6
	Std. Dev.	0	2.06 × 10^6^	2.54 × 10^7^	1.66 × 10^7^	2.05 × 10^7^	

**Figure 2:**
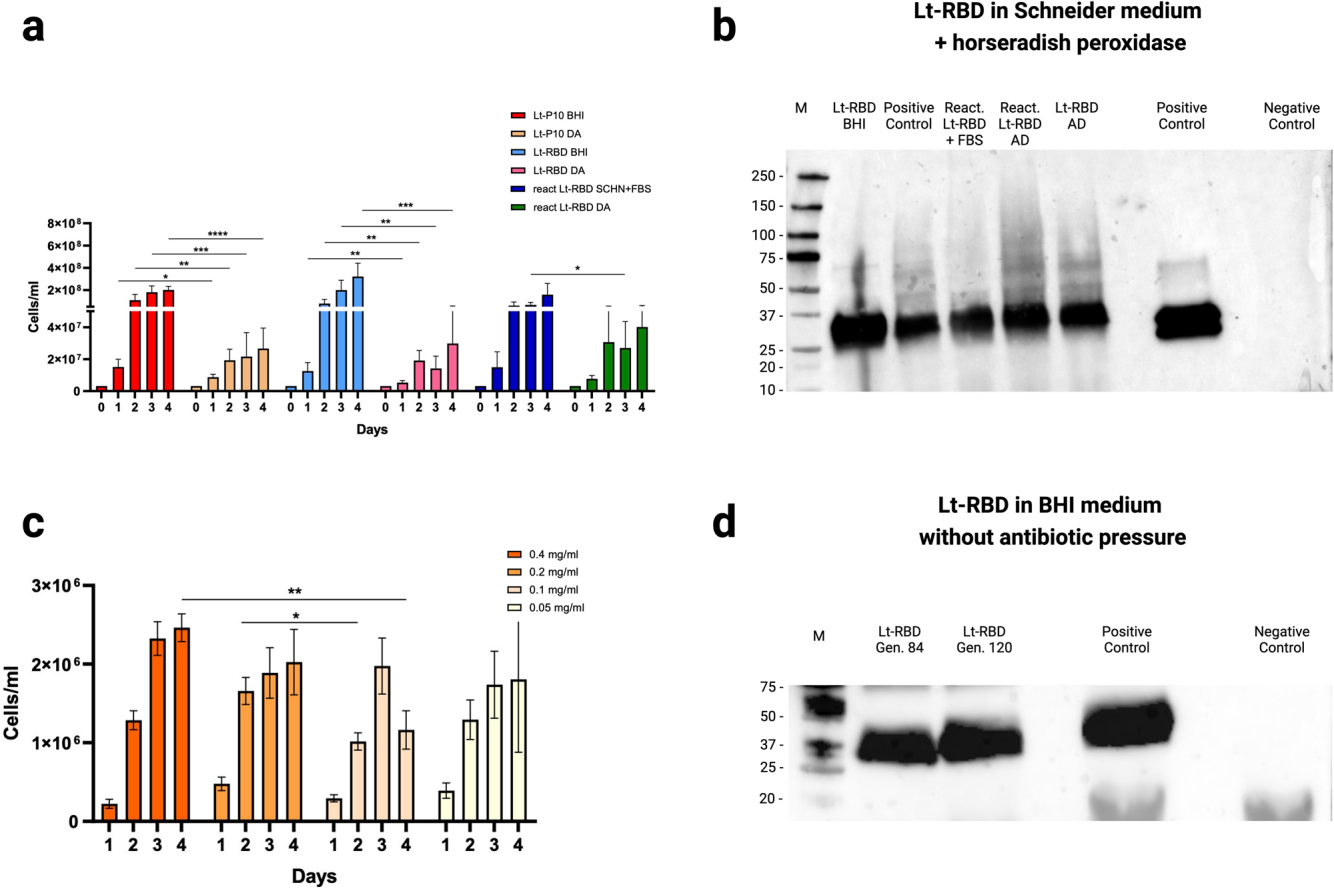
Comparison of cell growth in different media and evaluation of protein expression. (**a**) Growth curves of Lt-P10, Lt-RBD and reactivated Lt-RBD cultured in control media and in chemically-defined medium (Schneider + HRP) exploiting a direct adaptation strategy, for a 5-day period starting from a concentration of 3.2 × 10^6^ cell/ml. Bars show the mean ± SD. The significant differences were determined by t-test analysis. Differences were considered statistically significant when p < 0.05. The experiment was repeated three times. (**b**) RBD protein expression in Lt-RBD cells grown in Schneider + HRP (Direct Adaptation protocol) and in control medium (BHI and Schneider + FBS). (**c**) Growth curves of Lt-P10 cultured in Schneider at different Horseradish Peroxidase concentrations (0.4 mg/ml, 0.2 mg/ml, 0.1 mg/ml, 0.05 mg/ml) for a 5-day period starting from a concentration of 3 × 10^5^ cells/ml. Bars show the mean ± SEM. The significant differences were determined by two-way ANOVA (Tukey's multiple comparisons test). Differences were considered statistically significant when p < 0.05. The experiment was repeated three times. (**d**) RBD protein expression in Lt-RBD grown in BHI without the addition of the antibiotic nourseothricin up to 120 cell generations.

As shown in Fig. 2a, a significant difference in growth was observed between BHI and Schneider + HRP for both Lt-P10 and Lt-RBD at 24, 48, 72 and 96 h (for Lt-P10: p < 0.05, p < 0.01, p < 0.001 and p < 0.0001, respectively; for Lt-RBD: p < 0.01 for all time points except for 96 h with p < 0.001). In contrast, for reactivated Lt-RBD, no significant differences were observed between the serum supplemented medium and Schneider + HRP, at 24, 48 and 96 h; the only significant difference observed was at 72 h (p < 0.05) (Fig. 2a). In summary: (i) the reactivated Lt-RBD appears to display the best growth performance in the chemically-defined medium Schneider + HRP; (ii) the DA protocol guarantees optimal adaptation of *L. tarentolae* to Schneider + HRP (albeit with a lower growth efficiency compared to the classical BHI), and is also suited to obtain cells of the parasite suitable for long storage of *Leishmania* at − 80 °C. After 96 h, RBD protein expression in Lt-RBD and in the reactivated Lt-RBD grown in Schneider + HRP and in the conventional media (BHI and Schneider + FBS) was determined by Western blot analysis on cell pellets. For a proper comparison, the optical density (OD) from each culture was determined to compare the protein production from the same number of cells. The effective expression of RBD protein in all the conditions tested was confirmed: in the four samples tested, a unique band at 35 kDa, corresponding to the molecular weight of the heterologous protein RBD, was identified as the SARS-CoV-2 Spike RBD, using a specific antibody (Fig. 2b, Supplementary File 3). Then, the relative quantification of the protein was obtained with the Image Lab program (version 6.0.1). RBD protein band expressed by the reactivated Lt-RBD in Schneider + FBS was used as a reference band. As specified in Supplementary File 2, for the reactivated Lt-RBD, the estimated protein quantity in the chemically defined medium resulted 1.2 times more than that of the same strain in the conventional medium. Moreover, band intensity in Lt-RBD in BHI and in Schneider + HRP resulted to be respectively 2.2- and 1-48 folds than the reference band. In summary, RBD protein had been well expressed by Lt-RBD strains, in both the conventional medium and in the peroxidase-supplemented medium, as well as after storage at − 80 °C in Schneider + HRP (Supplementary File 2).

### Horseradish Peroxidase optimization

Different concentrations of HRP (0.40 mg/ml, 0.20 mg/ml, 0.10 mg/ml and 0.05 mg/ml) added to Schneider were tested on Lt-P10 culture monitoring cell survival and growth. At a concentration of 0.40 mg/ml and 0.20 mg/ml cells grew respectively 12.31- and 10.13-folds from the beginning of the week, resulting active elongated nectomonad, short nectomonad and metacyclic promastigotes (Table 3). On the contrary, cells grown with 0.1 mg/ml and 0.05 mg/ml concentration of HRP, resulted to grow less than the other two conditions (growth rate at 5.8 and 9.03 respectively) (Table 3). At the two lowest concentrations (i.e. 0.10 mg/ml and 0.05 mg/ml), cells were metacyclic nectomonads, not active or only with an active flagellum.

**Table 3:** Mean cell density, standard deviation (Std. Dev.) and growth rate of Lt-P10 cells cultured in Schneider's *Drosophila* Medium at different Horseradish Peroxidase concentrations.

Day	0.40 mg/ml		0.20 mg/ml		0.10 mg/ml		0.05 mg/ml	
	No cells/ml	Std. Dev.	No cells/ml	Std. Dev.	No cells/ml	Std. Dev.	No cells/ml	Std. Dev.
0	2 × 10^5^	0	2 × 10^5^	0	2 × 10^5^	0	2 × 10^5^	0
1	2.24 × 10^5^	1.66 × 10^5^	4.78 × 10^5^	2.45 × 10^5^	2.94 × 10^5^	1.24 × 10^5^	3.91 × 10^5^	2.76 × 10^5^
2	1.29 × 10^6^	3.40 × 10^5^	1.66 × 10^6^	4.89 × 10^5^	1.02 × 10^6^	3.13 × 10^5^	1.29 × 10^6^	7.12 × 10^5^
3	2.33 × 10^6^	6.02 × 10^5^	1.89 × 10^6^	9.11 × 10^5^	1.98 × 10^6^	1.01 × 10^6^	1.74 × 10^6^	1.20 × 10^6^
4	2.46 × 10^6^	4.98 × 10^5^	2.03 × 10^6^	1.17 × 10^6^	1.16 × 10^6^	6.89 × 10^5^	1.81 × 10^6^	2.62 × 10^6^
Growth rate	12.31		10.13		5.81		9.03	

As described in Fig. 2c, cell growth was significantly greater at the condition of 0.40 mg/ml compared to 0.10 mg/ml on day 4 (p < 0.05). Similarly, a statistically significant difference in Lt-P10 growth is observed between 0.10 and 0.20 mg/ml on day 2 (p < 0.01) (Fig. 2c).

Therefore, the addition of HRP at a final concentration of 0.40 mg/ml and 0.20 mg/ml to the chemically defined medium is recommended for cell adaptation without interfering with cell viability and morphology.

### Antibiotic removal

The technology used for the engineering of Lt-RBD strain exploits the chromosomal integration of a target gene in association with an antibiotic resistance gene. The use of the antibiotic nourseothricin allows thus the target gene maintenance and consequently the RBD protein expression^24,25^. However, for future medical and biotechnological applications of *L. tarentolae*, which implies its growth in industrial bioreactors, it is advisable the antibiotic resistance gene is removed^26–28^. As proved by other studies, engineered *Leishmania* appear to properly express recombinant proteins also in the absence of a selective antibiotic, in particular in case of a chromosomal integration of the target gene^10,29^.

Therefore, Western blot analysis was carried out to verify the RBD protein production of Lt-RBD culture maintained without the use of nourseothricin for 12 weeks (corresponding to 120 generations of the parasite). As reported in Fig. 2d, RBD protein was secreted after 120 generations without antibiotic pressure, suggesting that engineered clones of *Leishmania* can survive and secrete proteins also in these growth conditions. Other studies proposed the generation of auxotrophic strains of *Leishmania*, by knocking out an essential metabolic gene, to be complemented through the incorporation of the required gene function in the selective cassette (Fig. 2d and Supplementary File 4)^30^.

Overall, the search for a chemically defined medium for *Leishmania* maintenance resulted to be quite complex due to the necessary supplementation of specific nutrients such as iron. Three chemically defined media were tested as possible substitutes to BHI, with Schneider + HRP (0.40 mg/ml and 0.20 mg/ml) resulting to be the best alternative. In fact, this medium enables a good *L. tarentolae* replication without the use of animal components, also allowing the long storage and maintenance of *Leishmania* at − 80 °C. This chemically-defined medium can also be used to maintain engineered strains, such as Lt-RBD, guaranteeing the production of recombinant proteins.

As regards the engineered Lt-RBD, the heterologous protein production in absence of the selecting antibiotic was also verified, suggesting the possible removal of the antibiotic resistant gene in case of biotechnological applications of the cells at an industrial level.

In conclusion, the efforts to set up optimal medium and culture conditions for *L. tarentolae* cultivation will improve the usage of this protozoon for large scale protein expression, which is a prerequisite for translational application in antigens, vaccines or therapeutics production.

## Materials and methods

### *Leishmania tarentolae* strains

Promastigotes of *L. tarentolae* strain P10 (Lt-P10; Jena Bioscience, Jena, Germany) isolated from *Tarentola mauritanica* (Moorish gecko) and the engineered strain *L. tarentolae* constitutively expressing the SARS-CoV-2 receptor-binding domain (Lt-RBD) were used in this study^24,25^. Both strains were maintained under aerated conditions, in 25 cm^2^ cell culture flask at 26 °C in Brain Heart Infusion Broth Medium (BHI) (Sigma-Aldrich, St. Louis, MO, USA) supplemented with porcine hemin 5 μg/mL (Jena Bioscience, Jena, Germany) and 1% Penicillin–Streptomycin Solution (Euroclone, Milan, Italy). Lt-RBD culture was also supplemented with nourseothricin 100 µg/ml (Jena Bioscience, Jena, Germany) to select only the engineered clones expressing the heterologous protein RBD. The two strains were diluted in fresh BHI twice a week, monitoring their growth, motility and morphology using the optical microscopy.

### Chemically-defined media tested

**Figure 3:**
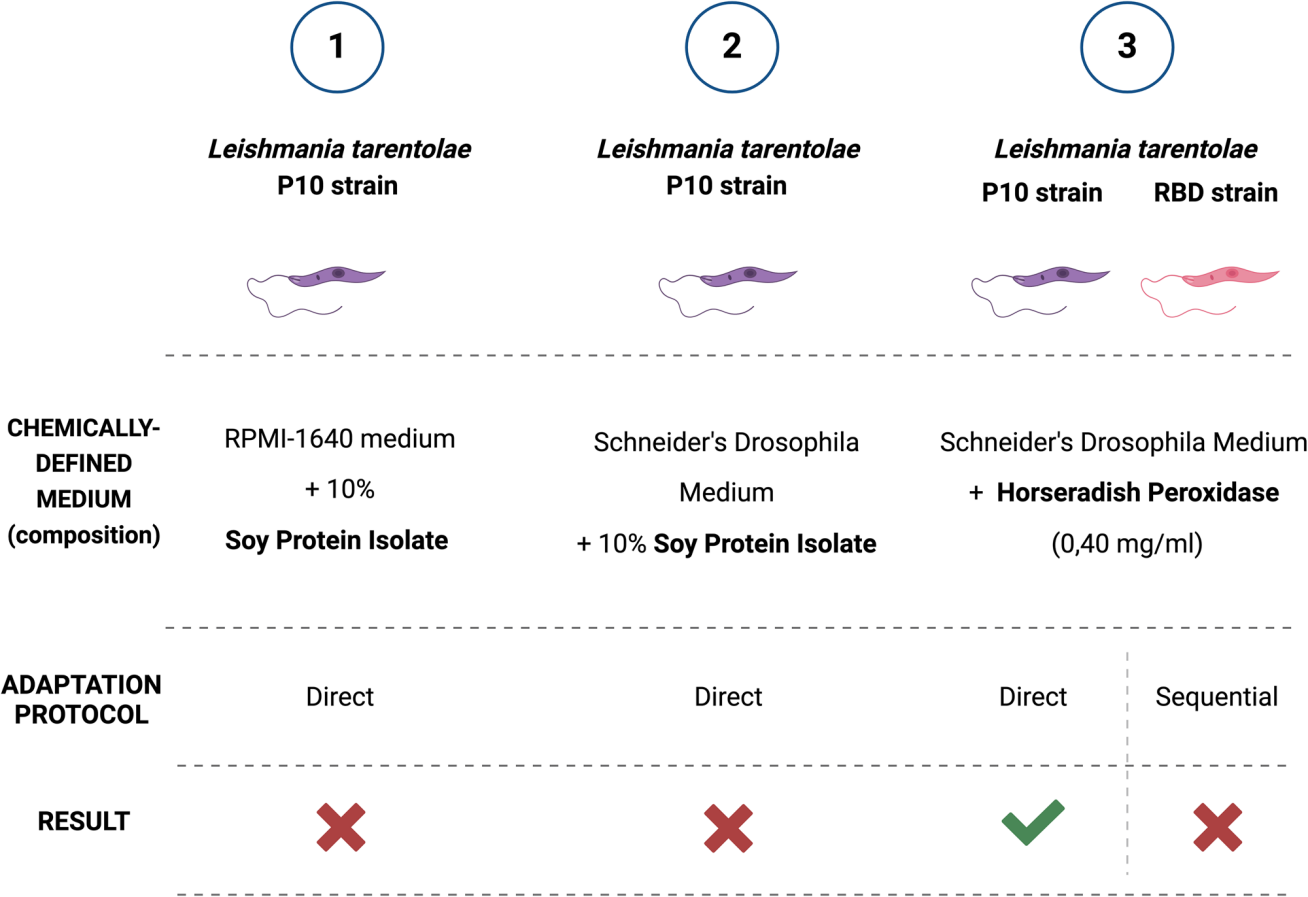
Summary of the chemically-defined media and the adaptation strategies (direct or sequential) tested on *Leishmania tarentolae*-P10 and on *Leishmania tarentolae*-RBD cultures.

Three chemically-defined media were tested for the culture adaptation of *L. tarentolae* promastigotes: (i) *RPMI-1640 Medium* + *soy protein isolate*; (ii) *Schneider's Drosophila Medium* + *soy protein isolate;* (iii) *Schneider's Drosophila Medium* + *Horseradish Peroxidase* (see Fig. 3).

#### RPMI-1640 Medium + soy protein isolate

*Leishmania tarentolae* culture was tested in RPMI-1640 Medium (RPMI) (Euroclone, Milan, Italy) supplemented with 10% sterile Soy Protein Isolate (SPI) (My Protein, Manchester, UK), 1% l-glutamine (Euroclone, Milan, Italy) and 1% Penicillin–Streptomycin solution (Euroclone, Milan, Italy). The same strain was also maintained in RPMI supplemented with 10% FBS (Euroclone, Milan, Italy), 1% l-glutamine (Euroclone, Milan, Italy) and 1% Penicillin–Streptomycin solution (Euroclone, Milan, Italy) and used as reference.

#### Schneider's *Drosophila* Medium + soy protein isolate

Schneider's *Drosophila* Medium (Schneider) (Thermo Fisher, Waltham, USA) was supplemented with 10% autoclaved SPI (My Protein, Manchester, UK), 1% Penicillin–Streptomycin solution (Euroclone, Milano, Italy) and tested for *L. tarentolae* maintenance. For comparison, Schneider supplemented with 10% FBS (Euroclone, Milan, Italy) and Penicillin–Streptomycin solution (Euroclone, Milan, Italy) was used.

#### Schneider's *Drosophila* Medium + Horseradish Peroxidase

Schneider (Thermo Fisher, Waltham, USA) added with Horseradish Peroxidase (HRP) (0.40 mg/ml; Thermo Fisher, Waltham, USA) was tested and the conventional BHI was used for comparison.

### Assays to adapt *Leishmania* strains to chemically-defined media

Two different strategies were tested for cell adaptation in the new media proposed: the direct adaptation strategy (DA) and the sequential adaptation strategy (SA) (see Fig. 3)^31^.

In the DA protocol, *Leishmania* cells maintained in animal-supplemented medium were inoculated directly in the tested chemically-defined medium. The concentration of the cells, motility and morphology were determined and compared with that obtained with the reference medium (serum-supplemented medium). By contrast, in the SA protocol cells were adapted from a serum-supplemented medium to a serum-free medium through several steps, characterized by a decreasing percentage of animal serum added (80%, 50%, 20%, 0%). For proper comparison, cells were subcultured twice a week with the same number of cells. These chemically-defined media were tested on both Lt-P10 and the engineered Lt-RBD to verify their potential use also for the cultivation of strains engineered for heterologous protein expression. However, the SA protocol was adopted only when the DA adaptation was successful. Similarly, the adaptation of the engineered Lt-RBD was tested only in media successfully tried out on Lt-P10.

The capability of the cells to grow in a chemically-defined medium was also evaluated after a long storage at − 80 °C. After a direct adaptation to Schneider + HRP, Lt-RBD was stored at—− 80 °C in this chemically defined medium following the protocol explained below. Briefly, 3.6 ml of cells (about 6 × 10^7^ cells/ml) were added to 1.2 ml of sterile glycerol and aliquoted in three cryovials. Each cryovial was then stored at—− 80 °C using Corning CoolCell LX (Corning, New York, USA). The correct adaptation of stored Lt-RBD in Schneider + HRP was verified as follows: Lt-RBD stocks were kept in ice for 20 min and then inoculated in 10 ml of Schneider + 20% FBS in a 25 cm^2^ cell culture flask at 26 °C. After two passages in this medium, cells were re-adapted in Schneider + HRP exploiting the DA protocol.

### Cell growth determination

Cell growth was monitored twice a week by optical microscopy observation, together with cell morphology and motility. Based on the morphology, cells were classified in three main categories: (1) elongated nectomonad; (2) short nectomonad and metacyclic promastigotes; (3) rounded metacyclic promastigotes and rounded promastigotes^32^. Then, *Leishmania* motility was taken into consideration comparing moving cells, static cells, and static cells with active flagellum. Finally, once the adaptation was completed, a slide from each condition was prepared and stained with Giemsa (Carlo Erba, Milan, Italy), following standard protocols, to compare cell morphology.

### Optimization of Horseradish Peroxidase concentration

After the adaptation of *Leishmania* to Schneider + HRP (0.40 mg/ml), the Lt-P10 strain was incubated in Schneider supplemented with four different concentrations of HRP to determine its optimal concentration: 0.40 mg/ml; 0.20 mg/ml; 0.10 mg/ml; 0.05 mg/ml.

A 12-well plate was used for the experiment and 2 × 10^5^ cells/ml were inoculated in each well with a final volume of 2 ml. As for the maintenance, the number of cells/ml, cell morphology and motility were monitored at the optical microscopy every day for a week.

### Western Blot analysis

The RBD protein production was verified in Lt-RBD culture, compared to the Lt-P10 strain (non-engineered) by Western Blot analysis, as described in Varotto-Boccazzi and colleagues^19,24^. Briefly, cell culture was centrifuged at 3000*g* for 10 min, then cell pellet was resuspended in loading buffer (Thermo Fisher Scientific, Massachusetts, US) and boiled for 5 min. The samples were loaded to Mini-PROTEAN TGX Stain-Free Protein (Bio-Rad Laboratories, Hercules, California, US) and, after the electrophoresis, proteins were transferred to a nitrocellulose membrane (Bio-Rad Laboratories, Hercules, California, US). After 5 min of incubation with EveryBlot Blocking Buffer (Bio-Rad Laboratories, Hercules, California, US), the membrane was incubated with SARS-CoV-2 Spike RBD antibody 1:5000 (Gene Tex, Irvine, California, US) (Bio-Rad Laboratories, Hercules, California, US) in blocking buffer for 2 h at room temperature. The membrane was then washed for three times with Phosphate-Buffered Saline (PBS) supplemented with 0.1% of Tween 20 (PBS-T) and incubated for 1 h with Goat anti-Rabbit IgG Secondary antibody HRP 1:30,000 (Thermo Fisher Scientific, Massachusetts, US). After three washes in PBS-T, the membrane was incubated with Clarity Western ECL Substrate (Bio-Rad Laboratories, Hercules, California, US) for 5 min and then analyzed with the ChemiDoc Touch Imaging System (Bio-Rad Laboratories, Hercules, California, US).

Subsequently, a quantitative analysis of RBD expression was carried out with Image Lab Software (version 6.0.1) (Bio-Rad Laboratories, Hercules, California, US).

### RBD expression in the absence of the antibiotic selection

The growth of the engineered Lt-RBD strain was verified in BHI without the addition of nourseothricin, the antibiotic for the selection of the engineered strain, for over 100 cell generations. Cells were thus monitored twice a week and the RBD expression was verified by Western Blot analyses, following the protocol described above, once a week for 12 weeks.

### Statistical analysis

Statistical analyses were performed by t-test or by two-way ANOVA (followed by Tukey's multiple comparisons test) using GraphPad Prism 8.0 (GraphPad, CA, USA). A p value less than 0.05 was considered statistically significant for all the experiments.

### Patent

The antigen Lt-RBD and its potential application have been described in the Patent N. IT 102021000004160.

### Supplementary Information

Supplementary Figures.

## Data Availability

All data generated and analyzed during this study are included in the manuscript and its supplementary Information files.
