# Supplementary material for: A novel chemically defined medium for the biotechnological and biomedical exploitation of the cell factory Leishmania tarentolae
Source: Sci Rep. 2024 Apr 26;14:9562. doi: 10.1038/s41598-024-60383-1 (PMC11053126; doi:10.1038/s41598-024-60383-1)
Supplement: Supplementary file 1 — Supplementary Figures. [file 41598_2024_60383_MOESM1_ESM.pdf]

A novel chemically defined medium for the biotechnological and biomedical exploitation of the cell factory *Leishmania tarentolae*

Giulia Maria Cattaneo[a], Ilaria Varotto-Boccazzi[a,b], Riccardo Molteni[a], Federico Ronchetti[a], Paolo Gabrieli[a,b], Jairo Alfonso Mendoza-Roldan[c], Domenico Otranto[c,d], Emanuele Montomoli[e,f], Claudio Bandi[a,b], Sara Epis[a,b]*

[a] Department of Biosciences, University of Milan, Milan, 20133, Italy

[b] Pediatric CRC 'Fondazione Romeo ed Enrica Invernizzi', University of Milan, Milan, 20157, Italy

[c] Department of Veterinary Medicine, University of Bari, Valenzano, 70010, Italy

[d] Department of Veterinary Clinical Sciences, City University of Hong Kong, SE Republic of China

[e] Department of Molecular and Developmental Medicine, University of Siena, Siena, 53100, Italy

[f] VisMederi, Siena, 53100, Italy

*corresponding author

e-mail: sara.epis@unimi.it

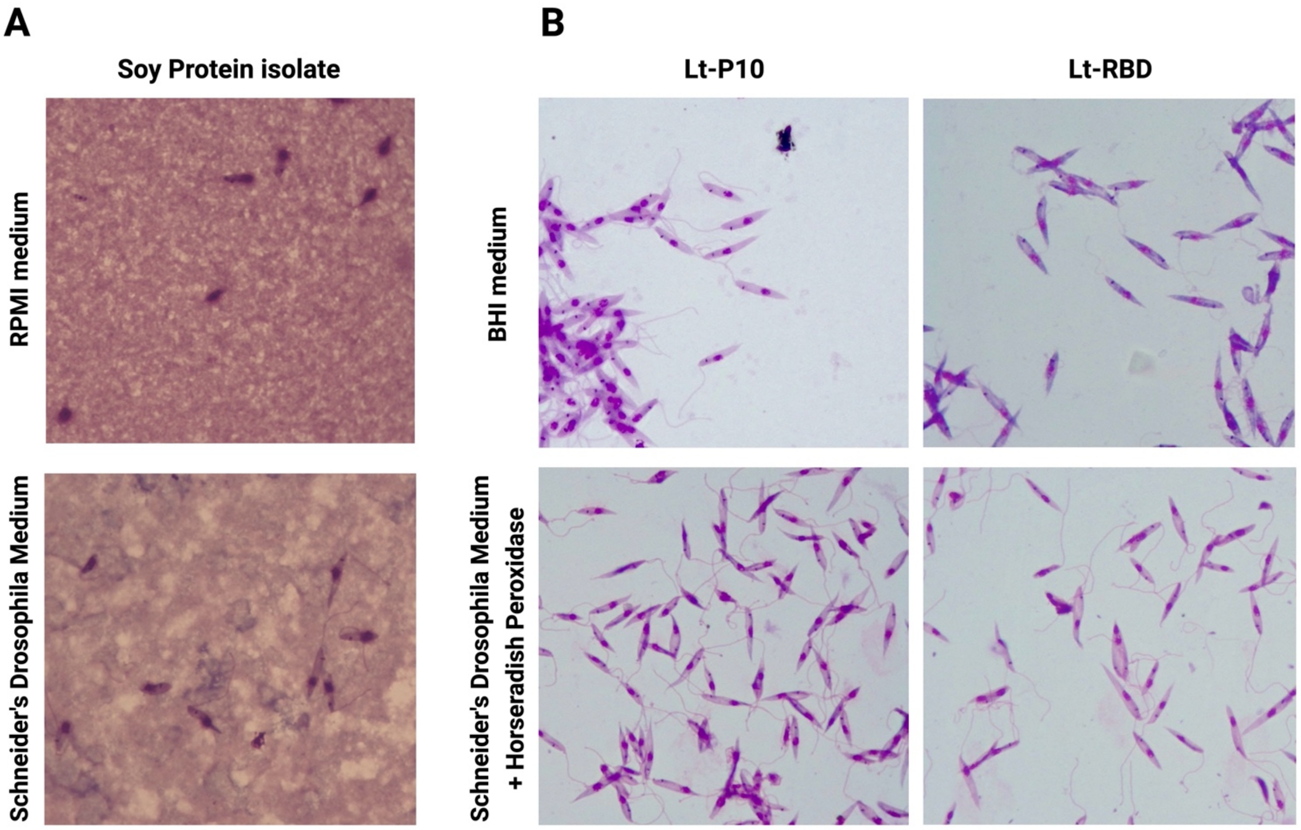

Supplementary File 1: Morphological evaluation of Lt-P10 and Lt-RBD promastigotes cultured in different chemically defined media. **(**A) Giemsa staining of Lt-P10 promastigotes cultured in RPMI medium + Soy Protein Isolate (SPI) and Schneider's *Drosophila* Medium + Soy Protein Isolate (SPI). (B) Giemsa staining of Lt-P10 and Lt-RBD in Schneider's *Drosophila* Medium + Horseradish peroxidase after a sequential adaptation protocol. Scale bar: 10µm.

Lane	Mol. Wt. (KDa)	Relative Front	Adj. Volume (Int)	Volume (Int)	Rel. Quant.	Band%	Lane %
RBD BHI	31,321352	0,758242	66014388	76199112	2,206901	100	91,691785
POSITIVE CONTROL	31,941373	0,750916	31453690	35979488	1,051516	100	70,643261
reactivated RBD SCHN+FBS	34,209709	0,725275	29912705	41346374	1	100	99,012212
reactivated RBD AD	35,230516	0,714286	37675350	58677034	1,25951	100	96,875583
RBD AD	35,57751	0,710623	44289360	61855500	1,48062	100	95,428296
POSITIVE CONTROL	33,876056	0,728938	25215898	90597239	0,842983	100	95,986225

Supplementary File 2: Comparison of RBD protein production in the engineered Lt-RBD and the reactivated Lt-RBD in Schneider's *Drosophila* Medium + FBS, BHI medium and Schneider's *Drosophila* Medium + Horseradish peroxidase (direct adaptation protocol). The relative quantification of RBD protein expression was performed using Image Lab Software (version 6.0.1). For a proper comparison, the same number of cells was considered from each condition.

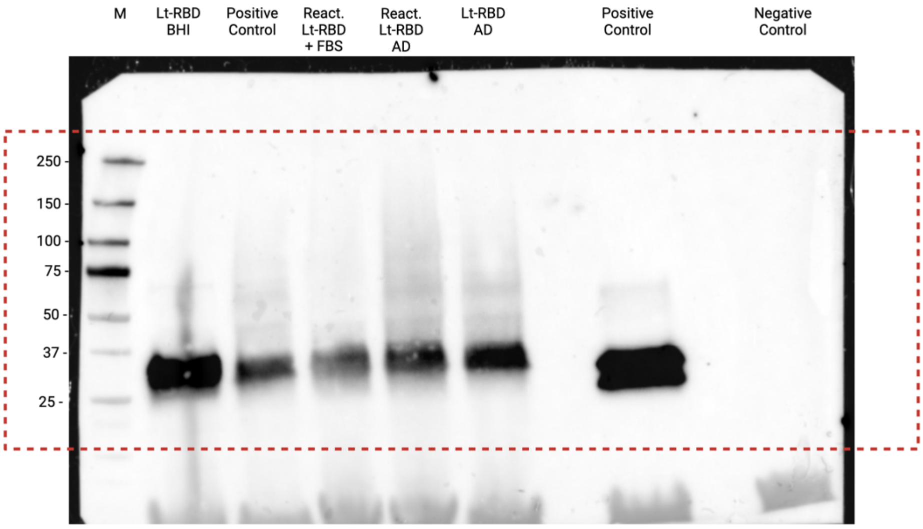

Supplementary File 3: Unedited image of Figure 3b. RBD protein expression in Lt-RBD and the reactivated Lt-RBD grown in Schneider + HRP (Direct Adaptation protocol) and in control media (BHI and Schneider + FBS). A band at 35 kDa, molecular weight of the heterologous protein RBD, was identified as RBD protein, using the specific SARS-CoV-2 Spike RBD antibody.

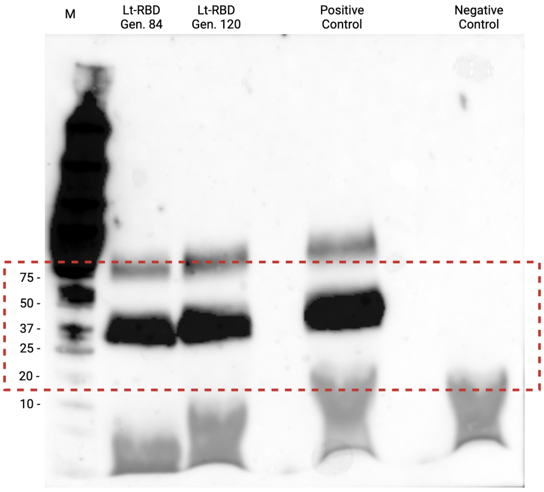

Supplementary File 4: Unedited image of Figure 3d. RBD protein expression in Lt-RBD grown in BHI without the addition of the antibiotic nourseothricin up to 120 cell generations. A band at 35 kDa, molecular weight of the heterologous protein RBD, was identified as RBD protein, using the specific SARS-CoV-2 Spike RBD antibody.
